# Bactericidal Effect of *Pseudomonas oryziphila* sp. nov., a Novel *Pseudomonas* Species Against *Xanthomonas oryzae* Reduces Disease Severity of Bacterial Leaf Streak of Rice

**DOI:** 10.3389/fmicb.2021.759536

**Published:** 2021-11-04

**Authors:** Ruihuan Yang, Shengzhang Li, Yilang Li, Yichao Yan, Yuan Fang, Lifang Zou, Gongyou Chen

**Affiliations:** ^1^School of Agriculture and Biology, Shanghai Jiao Tong University, Shanghai, China; ^2^State Key Laboratory of Microbial Metabolism, Shanghai Jiao Tong University, Shanghai, China

**Keywords:** *Pseudomonas oryziphila*, *Xanthomonas oryzae*, bactericidal effect, biocontrol agents, bacterial leaf streak of rice

## Abstract

*Pseudomonas* is a diverse genus of Gammaproteobacteria with increasing novel species exhibiting versatile trains including antimicrobial and insecticidal activity, as well as plant growth–promoting, which make them well suited as biocontrol agents of some pathogens. Here we isolated strain 1257 that exhibited strong antagonistic activity against two pathovars of *Xanthomonas oryzae*, especially *X*. *oryzae* pv. *oryzicola* (*Xoc*) responsible for the bacterial leaf streak (BLS) in rice. The phylogenetic, genomic, physiological, and biochemical characteristics support that strain 1257 is a representative of a novel *Pseudomonas* species that is most closely related to the entomopathogenic bacterium *Pseudomonas entomophila.* We propose to name it *Pseudomonas oryziphila* sp. nov. Comparative genomics analyses showed that *P. oryziphila* 1257 possesses most of the central metabolic genes of two closely related strains *P. entomophila* L48 and *Pseudomonas mosselii* CFML 90-83, as well as a set of genes encoding the type IV pilus system, suggesting its versatile metabolism and motility properties. Some features, such as insecticidal toxins, phosphate solubilization, indole-3-acetic acid, and phenylacetic acid degradation, were disclosed. Genome-wide random mutagenesis revealed that the non-ribosomal peptide catalyzed by LgrD may be a major active compound of *P. oryziphila* 1257 against *Xoc* RS105, as well as the critical role of the carbamoyl phosphate and the pentose phosphate pathway that control the biosynthesis of this target compound. Our findings demonstrate that 1257 could effectively inhibit the growth and migration of *Xoc* in rice tissue to prevent the BLS disease. To our knowledge, this is the first report of a novel *Pseudomonas* species that displays a strong antibacterial activity against *Xoc*. The results suggest that the *P. oryziphila* strain could be a promising biological control agent for BLS.

## Introduction

*Pseudomonas* species are gram-negative bacteria that are ubiquitous in soil, water, animals, and plant rhizosphere (Weller, 2007). Pseudomonads have the ability to grow rapidly and persist in plant rhizosphere, produce a wide range of secondary metabolites (i.e., antibiotics, siderophores, volatiles, and growth-promoting substances), and adapt to environmental stresses, which make them suitable as biocontrol agents of plant pathogens (Weller, 2007; Couillerot et al., 2009). Certain species of this genus have been demonstrated to be the effective biocontrol or growth-promoting agents. The 2,4-diacetylphloroglucinol (2,4-DAPG)-producing *Pseudomonas fluorescens* can suppress *Gaeumannomyces graminis*, a fungal pathogen, to control the take-all disease of wheat (Thomashow and Weller, 1988; Landa et al., 2003; Kwak et al., 2009). Besides significant suppression of the take-all disease, *P. fluorescens* strain 2-79 increased yields by an average of 17% in field trails (Weller, 2007). Some *Pseudomonas chlororaphis* strains are proficient biocontrol agents of many fungal, bacterial, and oomycete plant pathogens, which attributes to their ability to produce phenazines, pyrrolnitrine, hydrogen cyanide (HCN), siderophores, and volatile organic compounds (VOCs) (Anderson and Kim, 2018; Biessy and Filion, 2018). Phenazines and some VOCs are also involved in the induction of systemic resistance in plants (Raio and Puopolo, 2021). Therefore, there has been high increasing interest in *Pseudomonas* species for commercial and biotechnological applications.

Recent studies have shown that pseudomonads are also a resource reservoir for the control of plant bacterial diseases especially caused by plant pathogenic *Xanthomonas*. *Pseudomonas entomophila* is an entomopathogenic bacterium that is able to kill *Drosophila* larvae and adults. Its entomopathogenic property and hemolytic activity have been associated with insecticidal toxins and cyclic lipopeptides (Vodovar et al., 2005; Vallet-Gely et al., 2010). A recent study showed that *P*. *entomophila* harbors the bactericidal effect against *Xanthomonas citri* subsp. *citri* (*Xcc*), a causative agent of citrus canker (Villamizar et al., 2020). The saprophytic soil *Pseudomonas putida* has been reported to have the ability to inhibit three *Xanthomonas* bacteria *Xcc*, *Xanthomonas oryzae* pv. *oryzae* (*Xoo*), and *X*. *oryzae* pv. *oryzicola* (*Xoc*) (Sun et al., 2017). The *P*. *putida* group members including *Pseudomonas soli* and *Pseudomonas mosselii* produce a mixture of cyclic lipopeptides designated xantholysins that has the specific anti-*Xanthomonas* activity (Li et al., 2013; Pascual et al., 2014). Whether there are some unknown species in *Pseudomonas* genus that have the potential for controlling plant bacterial diseases caused by phytopathogenic *Xanthomonas* remains to be explored.

*Xoc* can infect the host rice causing bacterial leaf streak (BLS) that has gradually become the fourth major disease on rice in some rice-growing regions in southern China, resulting in a yield reduction of 10–30% in some severe cases (Nino-Liu et al., 2006; Ji et al., 2014). To date, no rice variety that is completely immune to *Xoc* was found (Xu et al., 2021). At present, all rice varieties commonly planted in China are susceptible to *Xoc*, even some hybrid rice varieties are highly susceptible. Currently, bactericides such as bismerthiazole or cupric pesticides are frequently used to control BLS in China (Xu et al., 2015; Pan et al., 2017), which has caused *Xoc* to develop resistance. Therefore, an effective and environmentally friendly biocontrol method for BLS is needed.

Our laboratory has been working to develop biological control methods to control BLS. As a part of our work on identifying useful bacterial resources, we screened 223 candidate isolates that exhibited apparent antagonistic activity against the *Xoc* wild-type strain RS105. In our previous studies, three *Bacillus* strains, *Bacillus velezensis* 504, *B. altitudinis* 181-7, and *Bacillus cereus* 512, have been reported to exhibit significantly inhibitory effects on water-soaked lesions caused by *Xoc* in rice leaves (Li et al., 2019; Li S. Z. et al., 2020). In this study, we characterized a *Pseudomonas* strain 1257 from the 223 candidate isolates. The phylogenetic, genomic, physiological, and biochemical characteristics demonstrated that strain 1257 is a representative of a novel *Pseudomonas* species, for which we propose to name *Pseudomonas oryziphila* sp. nov as its specific antibacterial activity against *Xoo* and *Xoc*. Genomic information showed that *P. oryziphila* 1257 is most closely related to the entomopathogenic bacterium *P. entomophila.* Genome-wide random mutagenesis revealed that a non-ribosomal peptide may be the major active compound involved in biocontrol agent of the *P. oryziphila* strain for BLS. To our knowledge, this is the first report of a novel *Pseudomonas* species that displays a specific antibacterial activity against *Xoc*.

## Materials and Methods

### Strains, Plasmids and Primers, Growth Conditions, and Plant Materials

The bacterial strains and plasmids are listed in Supplementary Table 1, and primers are listed in Supplementary Table 2. All *Xanthomonas* species strains were cultured in nutrient agar (NA) or nutrient broth (NB) medium at 28°C (Cai et al., 2017); *Escherichia coli* DH5α and EC100D were grown in Luria–Bertani (LB) medium at 37°C. Rice seeds (Yuanfengzhao) were provided by Dr. Youlun Xiao from the Institute of Plant Protection, Hunan Academy of Agricultural Sciences. Antibiotics were used at the following final concentrations (μg mL^–1^) as required: rifampicin (Rif), 75; kanamycin (Km), 25; gentamicin (Gm), 10; and spectinomycin (Sp), 25.

### Isolation of Biocontrol Strains and Antimicrobial Activity Assays

The rhizosphere soil samples from healthy plants were collected from 23 provinces in China. Briefly, a 10-g soil sample with several steel balls was added to a triangular conical flask with 90-mL sterilized water and then was shaken for 20 min at 28°C, 200 revolutions/min (rpm). A 1-mL suspension was diluted to three gradients of 10^–3^, 10^–4^, and 10^–5^ in sterilized water. The *Xoc* RS105 was used as an indicator, through inhibiting its growth to screen biocontrol strains. A 100-μL diluted solution was plated onto NA agar plates containing RS105. The plates were incubated at 28°C for 2 days. Colonies with antagonistic activity were transferred and purified and then were further confirmed by the Oxford Cup method. The inhibition zones were measured by Kirby–Bauer (KB) test method. Each inhibitory phenotype was repeated in triplicate. The antagonistic bacterial isolates were collected and stored at -80°C with glycerol (50%, vol/vol). Strain 1257 was isolated from the rhizosphere soil of cabbage collected on September 27, 2017 from Cungu village in Zhuanghang town, Fengxian District, Shanghai, China.

The antimicrobial activity of *P. oryziphila* 1257 was examined using *Xanthomonas* strains, non-*Xanthomonas* strains, and some fungal pathogen strains listed in Supplementary Table 1. All bacterial strains were inoculated into NB medium, followed by shaking at 28°C, 200 rpm for 12 h. The harvested cells were suspended and adjusted to a final concentration of OD_600_ = 2.0 using NB medium, and then the 200 μL solutions were added into the NA medium plates. A 50 μL (OD_600_ = 2.0) *P. oryziphila* 1257 solution was added into the Oxford Cup. The inhibition zones were measured using KB method and sterile water as the negative control. Three biological replicates were performed. The antifungal activity of *P. oryziphila* 1257 was further studied by confrontation–culture plating method according to the reported protocols.

### Phylogenetic Analysis Based on Multilocus Sequence and Average Nucleotide Identity Calculation

The morphology and physiological and biochemical characteristics of strain 1257 were identified by China Center for Type Culture Collection. Its *16S rRNA* gene sequence was amplified using universal primers of 27F and 1492R (Supplementary Table 2; Dorsch et al., 1992). The 50-μL reaction mixtures contained 5 μL of 10 × Ex Taq buffer (Mg^2+^ Plus) (20 mM), 4 μL of dNTP mixture (10 mM), 1 μL of each primer (10 μM), 1 μL of genomic DNA template (30–50 ng/μL), and 0.25 μL of Ex Taq DNA polymerase (TaKaRa,5 U/μL), with double-distilled water up to 50 μL. The polymerase chain reaction (PCR) amplifications were performed according to the following parameters: initial denaturation at 95°C for 8 min and then 32 cycles of 30 s of denaturation at 95°C, 30 s of annealing at 55°C, and 1.5 min of extension at 72°C, and a final extension at 72°C for 10 min. The 1,500-bp fragment was purified and sequenced in BioSune Biotech (Shanghai, China). The complete DNA sequence was used for blast search in the National Center for Biotechnology Information (NCBI) database. The multilocus sequence analysis (MLSA) phylogenetic tree was established and performed. The genome sequences for the other 15 representative *Pseudomonas* species were obtained from the NCBI database. The nucleotide sequences of 10 housekeeping genes *16Sr RNA*, *aroE*, *dnaA*, *guaA*, *gyrB*, *mutL*, *ppsA*, *pyrC*, *recA*, and *rpoB* were aligned using Muscle (version 3.8.425), and the unreliable comparison points were removed using Gblock (version 0.91b) to ensure that these sequences were suitable for phylogenetic analysis. A maximum likelihood–based phylogenetic method was performed with MEGE 7.0 software, with 1,000 bootstrap replicates; the internodes of branches indicated the percentage (Kumar et al., 2016). The average nucleotide identity (ANI) values among 16 genome sequences including *P. oryziphila* 1257 and other closest strains were calculated using the J Species WS Online Service (Richter et al., 2016). The relatedness of strains *P. oryziphila* 1257, *P. entomophila* L48, and *P. mosselii* CFML 90-83 was further determined by DNA–DNA hybridization as described by Fischer et al. (2011).

### DNA Extraction and Genome Sequencing

The genomic DNA of *P. oryziphila* 1257 was extracted using the Hipure bacterial DNA kit (Magen, Guangzhou, Guangdong, China); DNA quality and integrity were determined by using a Qubit Flurometer (Invitrogen, United States) and a NanoDrop Spectrophotometer (Thermo scientific, United States). The whole genome was sequenced using the Pacific Biosciences platform and the Illumina Miseq platform at Personalbio (Shanghai, China). The complete genome sequence of *P. oryziphila* 1257 was deposited in GenBank under accession number CP034338.1.

### Gene Family Construction and Collinearity Analysis

For comparative analyses of the orthologous and exclusive genes between 1257 and the other two closest genomes, the protein sequences of *P. oryziphila* 1257, *P. entomophila* L48, and *P. mosselii* CFML 90-83 were filtered to remove low-quality sequences based on length and percent stop codons in FASTA format. Then these proteomes were compared to each other based on an all-versus-all BLASTP alignment with an *E* value of 1e-10, I (Inflation) of 1.5 and at 70% identity. The BLASTP results are retrieved with the MCL program for clustering to construct gene families using OrthoMCL software (version 2.0.8). At last, through the Perl 5.8, DBI libraries to organize and count above clustering results were included.

Collinearity of the conserved and highly orthologous genomic regions were determined and plotted among *P. oryziphila* 1257, *P. entomophila* L48, and *P. mosselii* CFML 90-83 by using Mauve software (version 2.3.1) with default parameters (Darling et al., 2004). The colored, locally collinear blocks (LCBs) show the conserved and highly similar genomic regions. The white areas inside colored regions indicate sequence elements specific to one genome that are not aligned. The height of similarity profile is present inside each block. The colored lines that connect LCBs represent translocations of homologous regions. Blocks above or below the horizontal bar indicated regions that aligned in the forward or reverse orientation, respectively.

### The antiSMASH Analysis

The genomes of *P. oryziphila* 1257, *P. entomophila* L48, and *P. mosselii* CFML 90-83 were analyzed by antiSMASH 5.0 with web server^1^ to predict the putative secondary metabolite biosynthesis gene clusters (Blin et al., 2019). Detailed gene cluster information was obtained from the GenBank databases.

### Siderophore Production Detection

The chrome azurol S (CAS) assay was used for detecting the siderophore production according to the published methods (Schwyn and Neilands, 1987). Three strains of *P. oryziphila* 1257, *P. entomophila* L48, and *P. mosselii* CFML 90-83 were cultured in LB overnight, and then the suspensions of concentration with OD_600_ 2.0 in LB were obtained. A 5-μL solution was spotted on CAS agar plates, incubating at 28°C for 24 h, with three technical replicates.

### EZ-Tn5 Mutagenesis and Screening

Random mutagenesis was performed by electroporation of *P. oryziphila* 1257 competent cell with 1 μL of the EZ-Tn5 < R6Kγori/KAN-2 > Tnp transposome. The electroporated cells were immediately recovered with LB medium to the electroporation cuvette to 1-mL final volume. The medium was mixed gently by a pipette, transferred to a tube, and incubated on a 28°C shaker for 2 h to facilitate the cell outgrowth. Each 200 μL of the cells was plated on four plates containing Km and incubated at 28°C for 2 days. The colonies were moved individually to the NA agar medium containing *Xoc* RS105 and Km to screen the mutants that exhibited absolutely losing, apparently and partially attenuated antagonistic activity against *Xoc* RS105.

The genomic DNA from chosen mutants was digested by *Eco*RI and then were self-ligated by mixing 8 μL of the digested DNA with 1 μL of ligation buffer and 1 μL of T*4* DNA ligase (5 units; Thermo). The mixture was incubated at 22°C for 12 h and then inactivated at 70°C for 10 min. An aliquot of 5 μL of the ligation mixture was used to transform *E. coli* EC100D *pir*^+^ electrocompetent cells and then were plated on LB plates containing Km. Colonies were screened by amplified with the primers Tn5-F and Tn5-R in Supplementary Table 2. Insertion sites were confirmed by sequencing using the forward or reverse EZ-Tn5 < R6Kγori/KAN-2 > transposon-specific primers that were supplied in the kit.

### Construction of the Complemented Strains

Here we selected five mutants of 100-12, 56-11, 62-42, 62-27, and 92-23 to complement. Briefly, the open reading frame sequences with the promoter regions of *lgrD*, *carA*, *carB*, *purF*, and *serC* genes were amplified by PCR with primers listed in Supplementary Table 2. The corresponding PCR products were cloned into pML123, resulting in pML*-lgrD* by *Xba*I and *Hin*dIII digestion resulting in pML-*carA* and pML*-carB* by *Bam*HI and *Xba*I digestion, resulting in pML*-serC* by *Bam*HI and *Sac*I digestion, as well as resulting in pML*-purF* by *Hin*dIII and *Sac*I digestion. Correct recombinant plasmids were confirmed by PCR using the primers pML123-F and pML123-R listed in Supplementary Table 2. Subsequently, the recombinant plasmids were introduced into the corresponding mutants by electroporation.

### Biocontrol Assays

For biocontrol assays in rice fields, 10 leaves from the highly susceptible rice cultivar Yuanfengzao at booting stage were inoculated with *Xoc* RS105 (OD_600_ = 0.6) by needle injection. 1257 treatment (1257-Tre) meant that rice leaves were sprayed with 1257 (OD_600_ = 1.0) 12 h after inoculation with *Xoc* RS105 suspension. 1257 preventive treatment (1257-Pre) indicated that rice leaves were sprayed with 1257 12 h before inoculation with *Xoc* RS105 suspension. Three independent experiments were performed. The BLS disease severity under all treatments was investigated after 15 ays. For biocontrol assays in greenhouse, three leaves from 2-week-old Yuanfengzao were inoculated with *Xoc* RS105-Gus (OD_600_ = 0.6) by needleless syringe. The 1257-Tre and 1257-Pre indicated that rice leaves were injected with 1257 at 3 h after and before inoculation with *Xoc* RS105-Gus suspension, respectively. Three pots were injected in one leaf. Two independent experiments were performed. The BLS disease severity under all treatments was observed after 1, 3, 5, and 7 days. The inhibitory percentages (IPs) were calculated by the formula: IP = (1-lesions length of treatment/lesions length of control) × 100. The IP was calculated by using three technical replicates per assay. Student *t* test was used for significance (*p* < 0.05), and the statistical results were treated by GraphPad Prism 8 version.

### β-Glucuronidase Activity Assays

The β-glucuronidase (GUS) activity of *Xoc* RS105-Gus strains including staining and quantitative detection for each treatment in greenhouse was conducted as our previous methods (Li Y. L. et al., 2020; Zou et al., 2021).

## Results

### Isolation of Strain 1257 That Exhibits Strong Antagonistic Activity Against *Xanthomonas oryzae*

**FIGURE 1:**
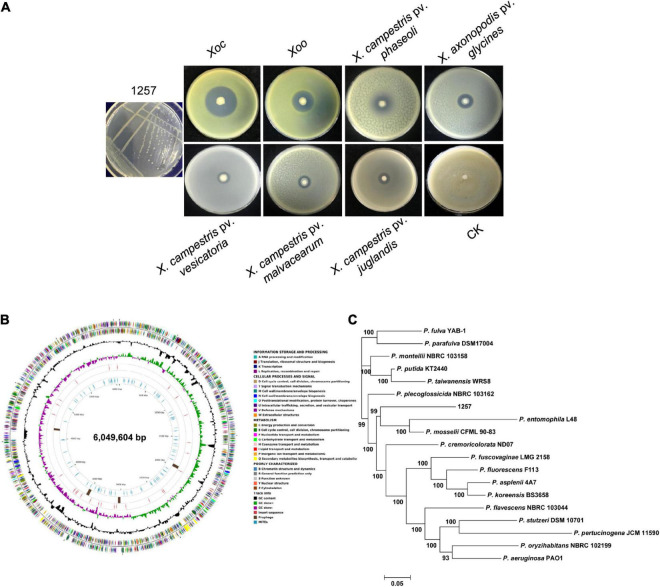
Isolation, identification, and antibacterial activity assays of strain 1257. **(A)** Colony morphology and antagonistic activity of strain 1257. From left to right represents strains of *Xoc* (RS105), *Xoo* (PXO99^*A*^), *X. campestris* pv. *phaseoli*, *X. axonopodis* pv. *glycines*, *X. campestris* pv. *vesicatoria*, *X. campestris* pv. *malvacearum*, and *X. campestris* pv. *juglandis* and control (CK). All tests were performed three times with similar results. **(B)** Circular genome map for strain 1257 using CG View. The outermost and second circles of all replicons indicate CDS on forward and reverse strands colored according to COG category. The third and fourth circles show G + C content and the G + C skew in green (+) and purple (−), respectively. The fifth circle shows the insertion sequences in red, putative prophage remnants in brown, and MITEs in blue. The scale is shown in the innermost circle. **(C)** The MLSA-based phylogenetic tree of strain 1257. The tree is based on 10 housekeeping genes. Maximum likelihood–based phylogenetic inference was performed with Mega 7. The bootstrap values of 1,000 replicates display the significance of each branch. Numbers at the branches indicate the confidence values of taxa clustered in the tree.

To screen beneficial bacterial resources to control BLS, we attained 223 bacterial isolates that displayed evident antibacterial activity against the *Xoc* wild-type strain RS105 from the 248 rhizosphere soil samples collected from 23 provinces in China. Among these isolates, we found that one strain, 1257, strongly inhibited *Xoc* RS105 (with an inhibition zone > 40 mm) and other eight *Xoc* strains isolated from Chinese major rice-growing regions (Figure 1A and Supplementary Figure 1). We found that 1257 also exhibited antagonistic effect against the *Xoo* wild-type strain PXO99^*A*^; however, the antibacterial activity against the *Xoc* strains by 1257 was significantly stronger than the *Xoo* strains (Figure 1A and Supplementary Figure 1). Further antibacterial activity assays showed that 1257 displayed a week antagonistic activity against other five Xanthomonads including *Xanthomonas campestris* pv. *phaseoli*, *Xanthomonas axonopodis* pv. *glycines*, *X. campestris* pv. *vesicatoria*, *X. campestris* pv. *malvacearum*, and *X. campestris* pv. *juglandis* (Figure 1A), and no evident inhibitory effect against *X. axonopodis* pv. *vasculorum, X. axonopodis* pv. *allii*, and *X. campestris* pv. *musacearum*, as well as other four non*-Xanthomonas* bacterial pathogens such as *Pseudomonas syringae* pv. *tomato* DC3000, *Ralstonia solanacearum*, *Burkholderia glumae*, and *Acidovorax citrulli* (data not shown). The antifungal activity assays showed that 1257 displayed no any inhibitory effect on five fungal pathogens including *Magnaporthe oryzae*, *Fusarium graminearum*, *Fusarium oxysporum*, *Botrytis cinerea*, and *Phytophthora capsici* (data not shown). These results suggest that 1257 exhibits specific inhibitory activity against *Xoc* and *Xoo*, two pathovars of *X. oryzae.*

### 1257 Is a Novel *Pseudomonas* Species Closely Related to *Pseudomonas entomophila*

The partial *16S rRNA* gene sequence of 1257 was amplified and aligned with the *16S rRNA* gene sequences that have been deposited in the NCBI database. The BLAST analysis indicated that 1257 belongs to the *Pseudomonas* genus; however, the *16S rRNA* gene sequence of 1257 exhibited more than 99% similarity with the corresponding sequences of *P. entomophila* L48 (99.54% similarity) and *P. mosselii* CFMT90-83 (99.48% similarity). To define the phylogenetic status of 1257, we sequenced the complete genome of 1257 (Figure 1B), which has been deposited in GenBank under accession number CP034338.1. The dendrogram deduced from 10 housekeeping genes (*16Sr RNA*, *aroE*, *dnaA*, *guaA*, *gyrB*, *mutL*, *ppsA*, *pyrC*, *recA*, and *rpoB*) using MLSA showed that 1257 located in a separate branch with the type strains of *P. entomophila* L48 and *P. mosselii* CFMT90-83 as its nearest neighbors (Figure 1C).

Further, we conducted an ANI analysis between 1257 and other 15 sequenced different species in the phylogenetic tree. All ANI values (ANIb and ANIm) between 1257 and individual species of the genus are in the range of 73.77 to 89.13% (Table 1), which is clearly below the threshold of 95% for species demarcation, indicating that 1257 was distinct to the type strains of all species. 1257 displayed an ANIb value of 86.55% with *P. entomophila* L48, and an ANIb value of 86.72% with *P. mosselii* CFMT90-83, confirming that 1257 should not be grouped into *P. entomophila* or *P. mosselii*. The DNA–DNA hybridization (DDH) value between 1257 and *P. entomophila* L48 was 34.10%, and the DDH value between 1257 and *P. mosselii* CFML 90-83 was 34.70%. These two values were less than the accepted species threshold of 70%. Together, these results suggested that 1257 is a novel species within the *Pseudomonas* genus, which is closely related to *P. entomophila* and *P. mosselii* species.

**TABLE 1:** ANI analyses between strain 1257 and other representative *Pseudomonas* species.

Reference genomes	Query genome of 1257	
	ANIb and [aligned nucleotides] (%)	ANIm and [aligned nucleotides] (%)
*Pseudomonas entomophila* L48 (DSM 28517)	86.55 [71.95]	89.10 [69.64]
*Pseudomonas mosselii* CFML 90-83 (DSM 17497)	86.72 [68.97]	89.13 [67.56]
*Pseudomonas taiwanensis* CMS (DSM 21245)	83.23 [63.20]	86.86 [53.72]
*Pseudomonas plecoglossicida* FPC951 (DSM 15088)	84.23 [62.41]	87.64 [56.13]
*Pseudomonas monteilii* strain 1 (DSM 14164)	83.92 [64.38]	87.21 [57.88]
*Pseudomonas putida* KT2440 (NBRC 14164)	83.96 [66.37]	87.36 [59.10]
*Pseudomonas oryzihabitans* L-1 (NBRC 102199)	73.77 [36.02]	83.94 [13.23]
*Pseudomonas fuscovaginae* LMG 2158 (ICMP 5940)	77.68 [49.92]	85.01 [27.95]
*Pseudomonas asplenii* 4A7 (DSM17133)	76.48 [54.33]	84.38 [24.32]
*Pseudomonas parafulva* CB-1 (DSM17004)	81.96 [57.30]	86.37 [44.99]
*Pseudomonas cremoricolorata* CC-8 (DSM 17059)	80.58 [50.98]	86.14 [38.63]
*Pseudomonas koreensis* Ps 9-14 (LMG 21318)	77.14 [54.28]	84.65 [28.37]
*Pseudomonas fulva* YAB-1 (DSM17717)	80.45 [56.12]	85.95 [38.51]
*Pseudomonas fluorescens* F113 (DSM50090)	76.53 [51.06]	84.47 [23.57]
*Pseudomonas aeruginosa* PAO1 (DSM50071)	76.10 [46.39]	84.36 [22.89]

*ANIb and ANIm values indicate the pairwise comparisons of given genomic sequences with the genome of strain 1257.*

**FIGURE 2:**
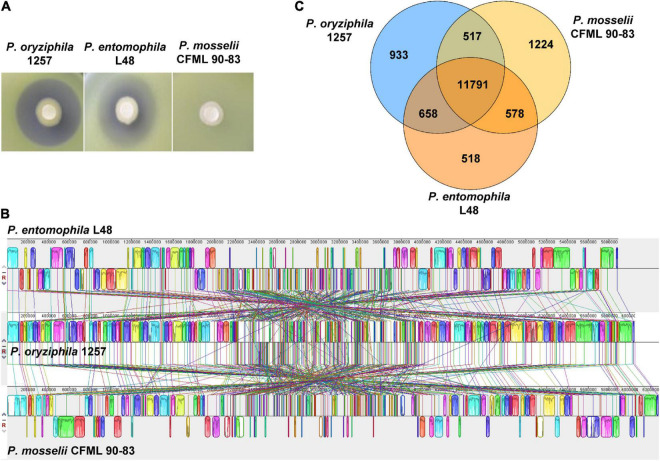
Comparative genomics analysis and antibacterial phenotypes of *P. oryziphila* 1257 with its closely related two *pseudomonas* species. **(A)** The inhibitory phenotypes of strains *P. oryziphila* 1257, *P. entomophila* L48, and *P. mosselii* CFML 90-83 against *Xoc* RS105. **(B)** Genome-to-genome alignment of *P. oryziphila* 1257, *P. entomophila* L48, and *P. mosselii* CFML 90-83 using a progressive mauve software with a window of 1,000 nucleotides and *P. oryziphila* 1257 as the reference genome. Boxes with the same color indicate the syntenic regions. Boxes below the horizontal line indicate inverted regions. Rearrangements are shown by colored lines. **(C)** Venn diagram showing the number of genes of orthologous CDSs shared and unique between three strains of *P. oryziphila* 1257, *P. entomophila* L48 and *P. mosselii* CFML 90-83.

Additional physiological and biochemical characteristics including enzyme activity, carbon source assimilation and acid production were tested by Biolog system using the Biolog GN2 microplates (Supplementary Table 3). The physiological and biochemical characteristics of *P. entomophila* L48 and *P. mosselii* CFML 90-83 were attained from the previous studies (Dabboussi et al., 2002; Mulet et al., 2012; Supplementary Table 3). These results showed that 1257 exhibited more than 21 phenotypic features similar to *P. entomophila* L48 and *P. mosselii* CFML 90-83, whereas 1257 differed from *P. entomophila* L48 and *P. mosselii* CFML 90-83 in the utilization of nine carbon sources especially in *N*-acetyl-D-glucosamine, D-arabitol, D-(+)-glucuronic acid, and 2,3-butanediol. 1257 displayed the same as *P. entomophila* L48 in urease activity and in the ability to use L-fucose, psicose, α-ketobutyric acid, and dimethyl succinate as carbon sources. 1257 differed from *P. entomophila* L48 in the capacity to use glycogen, which is negative for 1257 and *P. mosselii* CFML 90-83. Combined with the antagonistic phenotypes that *P. entomophila* L48 exhibited similar antibacterial activity against *Xoc* RS105 with 1257, but *P. mosselii* CFML 90-83 displayed no inhibitory effect on *Xoc* RS105 (Figure 2A), we concluded that 1257 is most closely related to *P. entomophila*.

Synthetically considering the phylogenetic, genomic, physiological, and biochemical characteristics, we propose strain 1257 as a representative of a novel *Pseudomonas* species, for which we propose to name *P. oryziphila* sp. nov. The type stain is *P. oryziphila* 1257^(T)^.

### Genome Features and Comparative Genomics Analysis

The genome of *P. oryziphila* 1257 is composed of a circular chromosome of 6,049,604 base pairs (Figure 1B) with an overall G + C content of 63.76% and 5,441 protein coding sequences (CDSs), 22 rRNA genes, 76 tRNA genes, and 73 other non-coding RNA genes, without plasmid, which is similar with *P. entomophila* L48, but distinct from *P. mosselii* CFML 90-83 (Table 2). The *P. oryziphila* 1257 genome is larger than that of the *P. entomophila* L48 and smaller than *P. mosselii* CFML 90-83. The collinearity analysis is consistent with the close relatedness among *P. oryziphila*, *P. entomophila*, and *P. mosselii* (Figure 2B). OrthoMCL analysis of the orthologous genes among the three genomes showed that 11,791 genes constitute the core genome, occupying 83.6 to 87.1% of each genome (Figure 2C); 89.6% (12,449 genes) and 88.6% (12,308 genes) of *P. oryziphila* 1257 genes have orthologs in the *P. entomophila* L48 and *P. mosselii* CFML 90-83, respectively (Figure 2C). Based on this analysis, we found that 933 genes (6.7%) are unique to the *P. oryziphila* 1257 genome.

**TABLE 2:** General features of genomes of *Pseudomonas oryziphila* (*Po*) 1257, *Pseudomonas entomophila* (*Pe*) L48, and *Pseudomonas mosselii* (*Pm*) CFMT90-83.

General features	*P*o 1257	*Pe* L 48	*Pm* CFMT90-83
Genome size (Mb)	6.05	5.89	6.28
GC content (%)	63.76	64.20	63.95
rRNA genes	22	22	22
tRNA genes	76	78	80
Other RNA genes	73	4	192
Coding density (%)	86.38	89.1	87.66
Protein coding sequences (CDS)	5,441	5,056	5,731
Plasmid	−	−	+

The *P. oryziphila* 1257 genome possesses most of the genes involved in the central metabolic pathways similar with *P. entomophila* L48 and *P. mosselii* CFML 90-83 including pentose phosphate pathway (PPP), the tricarboxylic acid cycle (TCA), and the Entner–Doudoroff (ED) pathway. We found no gene encoding a 6-phosphofructokinase present in the *P. oryziphila* 1257 genome. This is consistent with *P. entomophila* metabolism (Vodovar et al., 2006), indicating that *P. oryziphila* has an incomplete Embden–Meyerhof–Parnas pathway, and relies on a complete ED pathway for hexose utilization.

The *P. oryziphila* 1257 genome contains more than 25 transport-encoding genes, three of which encode TolC, HlyD, and PrtD related to type I secretion system. Notably, 21 genes encoding the type IV pilus system (T4P) were found in the genome of *P. oryziphila* 1257 (Supplementary Figure 2 and Supplementary Table 4), suggesting that *P. oryziphila* could move on solid surface through twitching or gliding motility. Three T4P genes, *pilN* (*chr*_*orf 05630*), *pilO* (*chr_orf 05631*), and *pilS* (*chr_orf 05634*), are involved in encoding type IVB pilus biosynthesis protein. Only seven T4P homologous genes in *P. oryziphila* 1257 were identified in the genome of *P. entomophila* L48, whereas 17 T4P orthologous genes were found in the genome of *P. mosselii* CFML 90-83 (Supplementary Table 4), indicating that *P. entomophila* and *P. mosselii* may have a dysfunctional T4P. A virB4 gene encoding the conjugative type IV secretory system protein was found in the genome of *P. oryziphila* 1257, but not in *P. entomophila* and *P. mosselii*. No gene encoding the type III secretion system (T3SS) was found in the genome of *P. oryziphila* 1257, but one gene encoding the T3SS effector HopPmaJ (T3SE) was found in *P. oryziphila* 1257, which is in agreement with the previous report in *P. entomophila* (Vodovar et al., 2006).

### Secondary Metabolite Biosynthetic Gene Clusters

Some potential second metabolites including toxins, antibiotics, and cyclic lipopeptides were found and are listed in Supplementary Table 5. *P. oryziphila* 1257 produces an insecticidal toxin AprA, an alkaline protease, which has been shown to be a key virulence factor in *P. entomophila* (Opota et al., 2011; Lee et al., 2018). Another gene rtx encodes the repeats-in-toxin (RTX) protein with cytotoxic and hemolytic activity (Vodovar et al., 2006; Zhang et al., 2014), which exhibits approximately 75% similarity with the homologs of *P. entomophila* and *P*. *mosselii*. We found that three TcdA-, TcdB-, and TccC-like insecticidal toxin complexes unique to *P. entomophila* are absent from *P. oryziphila* 1257. *P. oryziphila* 1257 possesses the HCN biosynthesis operon (*hcnABC*) but not the genes associated for the biosynthesis of 2,4-DAPG, phenazines, pyoluteorin, pyrrolnitrin, pyochelin, pyocyanine, and xantholysinABCD. We also detected several genes or gene clusters involved in plant–bacteria interactions in the genomes of *P. oryziphila* 1257, including the *iacR* gene involvement in the indole-3-acetic acid (IAA) degradation pathway, the *paa* gene involved in the phenylacetic acid (PAA) degradation pathway, indicating that *P. oryziphila* 1257 might help to balance the delicate IAA and PAA equilibrium in the rhizosphere. A unique *acoABC* operon involved in the biosynthesis of acetoin that has been known as one kind of VOC promoting plant growth was detected in the genome of *P. oryziphila* 1257. Similar to *P. entomophila* and *P*. *mosselii*, *P. oryziphila* also possesses the pyrroloquinoline quinone (PPQ) biosynthesis genes *pqqE*, *pqqD*, and *pqqB*, which were predicted to participate in phosphate solubilization. These results suggested that *P. oryziphila* 1257 may have the potential to promote plant growth.

**FIGURE 3:**
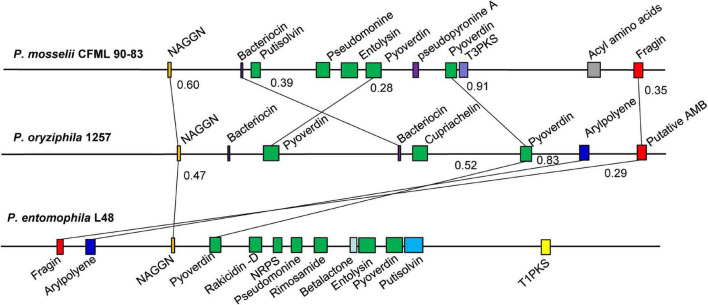
Comparison of the secondary metabolite biosynthesis gene clusters of *P. oryziphila*1257 with *P. entomophila* L48 and *P. mosselii* CFML 90-83. The number on the horizontal line represented the Jaccard index. Colinear regions are connected by black lines. NAGGN, *N*-acetylglutaminylglutamine amide; T3PKS, type III PKS; T1PKS, type I PKS (polyketide synthase); NRPS, non-ribosomal peptide synthetase cluster; AMB, L-2-amino-4-methoxy-*trans*-3-butenoic acid.

The antiSMASH analysis showed that the genome of *P. oryziphila* 1257 contains eight candidate gene clusters encoding a dipeptide *N*-acetylglutaminylglutamine amidc (NAGGN), four non-ribosomal peptide synthetase (NRPS), and two ribosomally synthesized antimicrobial peptides, and a pigment of the aryl polyene (APE) type (Figure 3 and Supplementary Table 6). The NAGGN cluster was predicted to synthesize NAGGN that is unusual dipeptide previously reported only in osmotically stressed *Rhizobium meliloti*, *P. fluorescens*, and *P. aeruginosa* PAO1 (Sagot et al., 2010). The two Bac 1 and Bac 2 clusters were predicted for the bacteriocin biosynthesis. However, these two clusters exhibited no apparent sequence similarity with the known strain. Three of four NRPS were predicted to be the siderophores biosynthetic gene clusters. The NRPS 1 and NRPS 3 are associated with the biosynthesis of pyoverdines. Nearly all fluorescent *Pseudomonas* species produce this yellow-green fluorescent that enable acquisition of Fe (III) ions from the surrounding environment (Gross and Loper, 2009). The genomes of *P. entomophila* L48 and *P. mosselii* CFML 90-83 also contain two independent clusters responsible for the biosynthesis of pyoverdines. In addition to pyoverdine, *P. entomophila* L48 can produce pseudomonine, an isoxazolidone siderophores, and pyochelin, a salicyl-capped siderophores. This is in agreement with our CAS agar diffusion assay, which showed that *P. entomophila* L48 can produce more total siderophores irrespective of chemical nature of the siderophores compared to *P. oryziphila* 1257 and *P. mosselii* CFML 90-83 (Supplementary Figure 4). Blast comparison showed that the NAGGN, NRPS 3, APE, and NRPS 4 cluster is conserved among the genomes of *P. oryziphila*, *P. entomophila*, and *P. mosselii*, whereas *P. entomophila* contains more candidate secondary metabolite biosynthetic gene clusters than that of *P. oryziphila* and *P. mosselii* (Figure 3). Combined with the antagonistic activity of three strains against *Xoc* RS105, we speculate that NRPS cluster but not the siderophores-producing NRPS cluster may be the target compound of *P. oryziphila* 1257 against *Xoc* RS105.

### Genome-Wide Identification of Antibacterial Mechanisms Against *Xoc*

**FIGURE 4:**
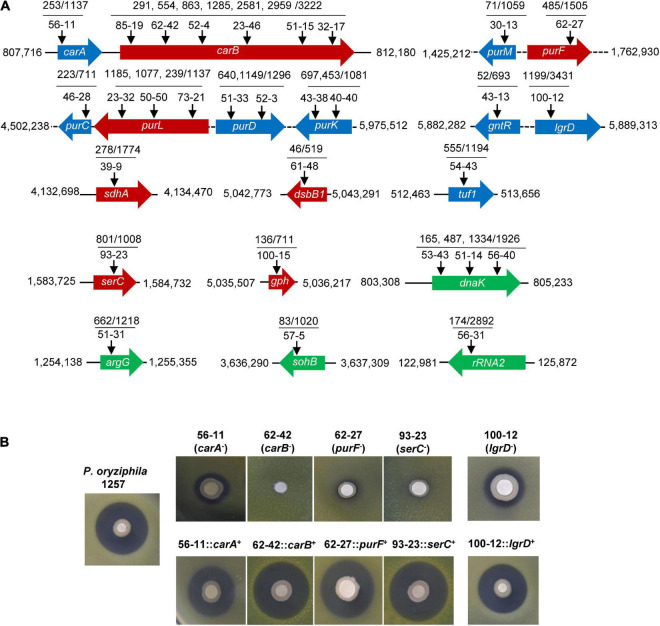
Genome-wide identification of the antibacterial functional genes of *P. oryziphila* 1257 against *Xoc* RS105. **(A)** Characterizations of Tn5 insertion mutants of *P. oryziphila* 1257. Arrows represent the Tn5 transposon insertion sites in 19 genes on the 1257 genome. Red, blue, and green arrow boxes indicate genes associated with absolutely losing, apparently, and partially attenuated antagonistic activity against *Xoc* RS105, respectively. **(B)** Antibacterial activity assays of the wild-type *P. oryziphila* 1257, *carA*, *carB*, *purF*, *serC*, and *lgrD* mutants and their corresponding complemented strains.

Our multiple attempts including utilization of ion exchange resin or optimization of the solvent were all failed to obtain the purified compound from 1257 that exhibited antibacterial activity against *Xoc* RS105. However, these attempts indicated that the active substance may be a strong polar compound. To elucidate the antibacterial mechanism of 1257, we used the transposon mutagenesis based on the EZ-Tn5 < R6Kγori/KAN-2 > Tnp transposome system to screen functional genes associated with antibacterial active compounds. Among the 10,080 mutants, we screened 30 mutants that exhibited absolutely lacking, apparently or partially attenuated antagonistic activity toward *Xoc* RS105 (Supplementary Figure 3). Southern blot analysis indicated that all mutants carried only a single copy of the transposon (data not shown). The Tn5 transposon insertions were mapped to 19 genes including *carAB*, *purMF*, *purCLDK*, *gntR*-*lgrD*, *sdhA*, *dsbB1*, *tuf1*, *serC*, *gph*, *dnaK*, *argG*, *sohB*, and *rRNA2* located in 13 different districts in the genome of 1257 (Figure 4A). In the *carA-carB*, *purM*-*purF*, *purC*-*purK*, and *gntR*-*lgrD* regions, transposons were inserted more frequently. Six independent insertions in the *carB* gene and three independent insertions in the *purL* gene resulted in loss of antagonistic activity of these mutants against *Xoc* RS105. One insertion in the *purF*, *sdhA*, *dsbB1*, *serC*, and *gph* genes also made 1257 lose the antagonistic activity. Two independent insertions in the *purD* and *purK* genes and one insertion in the *carA*, *purM*, *purC*, and *tuf1* genes reduced the antagonistic properties of 1257 against *Xoc* RS105. We constructed the full functional segments of the *carA*, *carB*, *purF*, and *serC* genes and introduced the relative plasmids into the corresponding insertion mutants. The complemented strains were found to restore the antagonistic activity against *Xoc* RS105 to the wild-type levels (Figure 4B), indicating the critical role of these genes in biosynthesis of active compounds antagonizing *Xoc* RS105.

**FIGURE 5:**
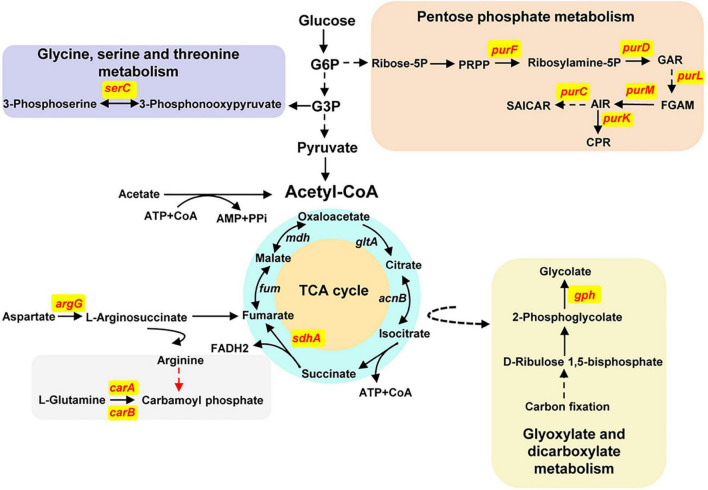
Schematic diagram of KEGG analyses about the antibacterial activity–associated genes of *P. oryziphila* 1257. The solid arrows indicate direct metabolites, and the dashed arrows indicate indirect metabolites. The red words indicate the genes associated with absolutely losing and apparently attenuated antagonistic activity against *Xoc* RS105. TCA cycle, tricarboxylic acid cycle; G6P, glucose-6-phosphate; G3P, glycerate-3-phosphate; ribose-5P, ribose-5-phosphate; PRPP, 5-phosphoribosyl diphosphate; ribosylamine-5P, 5-phosphoribosylamine; GAR, 5′-phosphoribosylglycinamide; FGAM, 2-(formamido)-N1-(5′-phosphoribosyl) acetamidine; AIR, aminoimidazole ribotide; SAICAR, 1-(5′-phosphoribosyl)-5-amino-4-(*N*-succinocarboxamide)-imidazole; CPR, 5-carboxyamino-1-(5-phospho-D-ribosyl) imidazole.

To define the biological process involved by the function genes mentioned previously, we exerted a Kyoto Encyclopedia of Genes and Genomes (KEGG) analysis (Figure 5). The *carA* and *carB* genes encode the carbamoyl phosphate synthetase that catalyzes the synthesis of carbamoyl phosphate (CP), a precursor of arginine and pyrimidines metabolism. ArgG encoded by the *argG* gene is an argininosuccinate synthetase responsible for producing argininosuccinic acid from aspartate. The *sdhA* gene encodes a subunit A of succinate dehydrogenase that is a key enzyme in the TCA cycle. The six *pur* genes encoded PurF, PurD, PurL, PurM, PurC, and PurK, which are metabolic enzymes in the PPP, converting ribose-5P from the glycolytic pathway (PP) into 5-carboxyamino-1-(5-phospho-D-ribosyl) imidazole (CPR). Together, these analyses indicated that CP and CPR may be the important precursors for synthesis of the activate compounds and also provided some important evidences for further identification of the target compounds.

Three independent insertions have been shown in *dnaK* encoding a chaperone protein foldase Dnak that enable the RNA polymerase to sustain bacterial life in response to the stringent response (Kim et al., 2021). Another two Tn5 insertions in the *sohB* gene encoding a periplasmic serine protease (ClpP class) and *rRNA2* gene encoding the 23s_rRNA, respectively, resulted in partially impaired antagonistic activity of the mutants against *Xoc* RS105, indicating that posttranscriptional mechanisms may be involved in the modulation of genes associated with biosynthesis of active compounds.

In particular, two individual insertions have been found in the *gntR* and *lgrD* genes that located in the NRPS 4 cluster exhibiting 40% similarity with the L-2-amino-4-methoxy-*trans*-3-butenoic acid (AMB) biosynthetic gene cluster from *P. aeruginosa* PAO1 by the antiSMASH analysis. However, the *gntR* gene encodes a GntR family transcriptional regulator, and the *lgrD* gene encodes a non-ribosomal peptide synthethase of linear gramicidin synthase subunit D. The *gntR* and *lgrD* insertion mutants (43-13 and 100-12, respectively) exhibited significantly reduced antibacterial activity against *Xoc* RS105 when compared with the wild-type *P. oryziphila* 1257, whereas the complemented strain of *lgrD* insertion mutant nearly restored the antibacterial activity to the wild-type levels (Figure 4B). These results indicated that the non-ribosomal peptide catalyzed by LgrD may be a major active compound of *P. oryziphila* 1257 against *Xoc* RS105.

Sequence alignments showed that proteins encoded by the 19 genes mentioned previously in *P. oryziphila* 1257 exhibited more similarity with the homologs in *P. entomophila* L48 than *P. mosselii* CFML 90-83 (Supplementary Table 7), further supporting that *P. oryziphila* 1257 is most closely related to *P. entomophila* species.

### Biocontrol Effect of *Pseudomonas oryziphila* 1257 in Bacterial Leaf Streak

**FIGURE 6:**
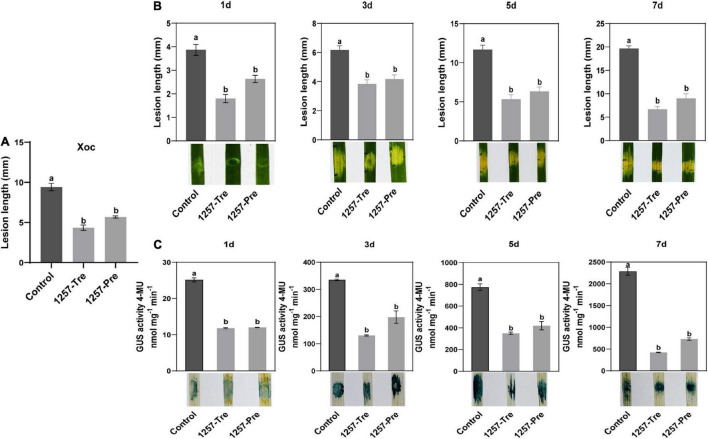
Biocontrol effect of *P. oryziphila* 1257 in BLS of rice. **(A)** Lesion lengths of the susceptible rice cultivar Yuanfengzao inoculated by *P. oryziphila* 1257 and *Xoc* RS105 at 15 days in rice field trials. 1257 treatment (1257-Tre) meant that rice leaves were sprayed with 1257 12 h after inoculation with *Xoc* RS105 suspension. 1257 preventive treatment (1257-Pre) indicated that rice leaves were sprayed with 1257 12 h before inoculation with *Xoc* RS105 suspension (1257-Tre). **(B)** Lesion lengths of the rice cultivar Yuanfengzao inoculated by *P. oryziphila* 1257 and *Xoc* RS105 at 1, 3, 5, and 7 days in greenhouse. The 1257-Tre and 1257-Pre indicated that rice leaves were injected with 1257 after and before 3 h inoculation with *Xoc* RS105-Gus suspension, respectively. **(C)** Dynamic population of *Xoc* RS105-Gus in rice leaves monitored by the GUS quantification detection and histochemical staining at 1, 3, 5, and 7 days after inoculation. *Xoc* RS105 and Xoc RS105-Gus were adjusted to OD_600_ = 0.6, and *P. oryziphila* 1257 was adjusted to OD_600_ = 1.0. Different treatments were compared using the least significant difference test method; error bars indicate standard deviation, and the different letters indicate significant differences (*p* < 0.05). Experiments were repeated more than two times and showed the similar results.

To investigate the biocontrol efficiency of *P*. *oryziphila* 1257 in BLS caused by *Xoc* RS105, we executed a field trial experiment using a highly susceptible cultivar Yuanfengzao as follows: *Xoc* RS105 only (control), rice leaves sprayed with 1257 12 h after inoculation with *Xoc* RS105 suspension (1257-Tre) and rice leaves sprayed with 1257 12 h before inoculation with *Xoc* RS105 suspension (1257-Pre). We fist tested the appropriate concentration of 1257 and found that 1257 as OD_600_ of 1.0 did not influence the morphology and growth of the rice plants during 15 days in our field trial experiment. Therefore, we used this concentration in all our biocontrol assays. The BLS disease severity under all treatments was investigated after 15 days. Compared with the control treatment, the 1257-Tre and 1257-Pre treatments significantly reduced the severity of BLS in paddy fields with relative control efficiencies of 53.9 and 39.7%, respectively (Figure 6A). This demonstrated that the 1257-Tre treatment exhibited efficient biocontrol of BLS in the field.

Further, we carried out a greenhouse trial experiment using a *Xoc* RS105-Gus strain in which the wild-type *Xoc* RS105 carried a *hrcC*-*uidA* reporter plasmid as the control treatment. The 1257-Tre and 1257-Pre indicated that rice leaves were injected with 1257 3 h after and before inoculation with *Xoc* RS105-Gus suspension, respectively. The BLS disease severity under all treatments was observed after 1, 3, 5, and 7 days. On the third and fifth day, significantly reduced water-soaked lesions were observed on the leaves of Yuanfengzao by the 1257-Tre and 1257-Pre treatments compared to the control (Figure 6B). The relative control efficiencies by the 1257-Tre and 1257-Pre treatments were 66.10% and 54.30% on day 7, respectively. The GUS histochemical staining with the corresponding leaves showed similar result.

Given that the depth of GUS staining in rice leaves was dependent on bacterial multiplication, we used the quantifiable GUS measurement to determine the growing bacterial population in rice leaves by the control, 1257-Tre, and 1257-Pre treatments using our new method, which is more rapid and accurate than the conventional bacterial number counting (Zou et al., 2021). The quantitative GUS assays showed that the *Xoc* RS105 population-related GUS activity was dramatically lower in the rice leaves treated by 1257 regardless of the 1257-Tre or 1257-Pre treatment than that treated by the control at 1, 3, 5, and 7 days (Figure 6C). Taken together, these results indicate that 1257 could effectively inhibit the growth and migration of *Xoc* RS105 in rice tissue to prevent the BLS disease, making it a promising biological control agent for BLS.

## Discussion

*Xoo* and *Xoc* cause bacterial leaf bright and BLS of rice, respectively, which are two major bacterial diseases of rice in some Asian rice-growing regions (Nino-Liu et al., 2006). In this study, we identified a novel *Pseudomonas* species, *P. oryziphila*, which has the capacity to inhibit *Xoo* and *Xoc* and especially inhibit the growth and migration of *Xoc* in rice tissue to prevent the BLS disease. Genomic information revealed that *P. oryziphila* may have potential to kill insects, solubilize phosphate, move dependently on the T4P system, and degrade IAA and PAA, indicating that it is a versatile bacterium. Our findings collectively indicate that a non-ribosomal peptide may be the major active compound involved in this biocontrol of BLS. The new discovery of *P. oryziphila* also provides more microbial resources for biocontrol of bacterial diseases of rice.

*Pseudomonas* is a diverse genus with more than 200 different species, whereas many new isolates are being classified as a novel species. In 2019, for instance, 16 novel *Pseudomonas* species were described from different sources such as tree bark, sewage, or raw milk (Hofmann et al., 2021). Our results support that strain 1257 is a novel *Pseudomonas* species, which is more closely related to *P. entomophila* than *P. mosselii*. In this study, we used a polyphasic approach including genotypic and phenotypic analyses to characterize this novel species. At first, 1257 could be assigned to the genus *Pseudomonas* by the *16S rRNA*-based dendrogram, but not to any validly named species because the *16S rRNA* gene sequence of 1257 exhibited more than 99% similarity with the one of the type strains of *P. entomophila* L48 and *P. mosselii* CFMT 90-83. Further MLSA, ANI, and DDH analyses showed that 1257 located in a separate branch with *P. entomophila* and *P. mosselii*; however, the relatedness of this species could not be defined, indicating that sometimes genomic analyses such as MLSA, ANI, and DDH values are not enough to define the close relative relationship of some species. A similar case about the taxonomic characterization of *P*. *cremoris* sp. nov. was reported in the previous study (Hofmann et al., 2021). For example, ANIm comparisons indicated that the type strain WS 5106 was a novel species within the *P*. *fluorescens* subgroup, but the pairwise ANIm values of 90.1 and 89.8% showed that WS 5106 was most closely related to *Pseudomona*s *nabeulensis* CECT 9765^*T*^ and *Pseudomona*s *kairouanensis* CECT 9766^*T*^ (Hofmann et al., 2021). Therefore, some phenotypic analyses such as physiological and biochemical characteristics, or antagonistic activity, are necessary for further classification status. Our additional phenotypic analyses including enzyme activity, carbon source assimilation, and acid production showed that 1257 exhibited more similar phenotypic features with *P. entomophila* L48 than *P. mosselii* CFML 90-83, supporting that 1257 is most closely related to *P. entomophila*. We designated this novel species as *P. oryziphila* sp. nov., given its specific antibacterial activity against *Xoo* and *Xoc*, which is similar with *P. entomophila* that was named for its unique entomopathogenic property (Vodovar et al., 2005; Mulet et al., 2012).

The collinearity analysis that more than 85% of *P. oryziphila* 1257 genes have orthologs in the *P. entomophila* L48 and *P. mosselii* CFML 90-83 is consistent with the close relatedness among *P. oryziphila*, *P. entomophila*, and *P. mosselii.* Interestingly, comparative genomics analysis revealed that *P. oryziphila* contains a set of T4P biogenesis-associated genes (21 genes) including *pilA* encoding the major pilin protein (Maier and Wong, 2015); the *pilM*, *pilN*, *pilO*, and *pilP* genes encoding the alignment complex (PilM, PilN, PilO, and PilP) (Gold et al., 2015); the *pilT* and *pilC* genes encoding the motor (PilT1, PilT2, and PilC) (McCallum et al., 2019); the *pilQ* genes encoding the outer membrane (OM) pore complex PilQ1 or PilQ2; other genes such as *pilD* encoding a pre-pilin peptidase PilD; *pilS* encoding one of the two-component system PilS (Kilmury and Burrows, 2018); and the minor pilins FimT, PilV, and PilE-encoding genes (Treuner-Lange et al., 2020). These findings indicated that *P. oryziphila* may have a functional T4P. However, only seven T4P homologous genes were found in *P. entomophila* L48, whereas 17 T4P orthologous genes were found in *P. mosselii* CFML 90-83, indicating that T4P biogenesis-associated genes exhibit a high degree of variability among *P. oryziphila*, *P. entomophila*, and *P. mosselii*.

The biocontrol properties of *Pseudomonas* species are largely dependent on its secondary metabolites such as toxins, lipopeptides, polyketides, fatty acids, and phenazines (Gross and Loper, 2009). *P*. *oryziphila* 1257 contains the insecticidal toxin AprA and hemolytic RTX toxin, which is in agreement with the previous finding in *P. entomophila* L48 (Vodovar et al., 2006). This indicates that *P*. *oryziphila* stain could be a versatile bacterium capable of inhibiting *X. oryzae* and killing insects or *Drosophila melanogaster*. A similar study has been reported that *P. entomophila* JS2 displayed a clear antibacterial effect against *Xcc* 306, the causal agent of citrus canker (Villamizar et al., 2020). We found that *P. oryziphila* 1257 possesses the HCN biosynthesis operon (*hcnABC*) but not the genes associated for the biosynthesis of 2,4-DAPG, phenazines, pyoluteorin, pyrrolnitrin, pyochelin, pyocyanine, and xantholysinABCD. Xantholysins, a family of lipodepsipeptides produced by some *P*. *putida* and *P*. *soli* strains, exhibits *Xanthomonas*-inhibitory activity, including *Xoo*-antagonistic activity (Vallet-Gely et al., 2010; Pascual et al., 2014). Although *P*. *soli* is closely related to *P. entomophila* and *P. mosselii*, the xantholysin-encoding genes are distinguishable targets between two novel Pseudomonas species of *P*. *oryziphila* and *P*. *soli*. We also detected several genes or gene clusters including the *iacR* gene (IAA degradation pathway) and the *paa* gene (PAA degradation pathway) in the genomes of *P. oryziphila* 1257, indicating that *P. oryziphila* 1257 might help to balance the delicate IAA and PAA equilibrium in the rhizosphere. The *acoABC* operon putatively participated in the biosynthesis of acetoin, one kind of VOC promoting plant growth (Wu et al., 2019), and PPQ biosynthesis genes *pqqE*, *pqqD*, and *pqqB* predicted to participate in phosphate solubilization were found in the genome of *P. oryziphila* 1257. These findings suggested that *P. oryziphila* 1257 has the potential to promote plant growth, but whether *P. oryziphila* 1257 is a plant growth–promoting rhizobacteria strain needs more experimental evidences.

Our unsuccessful attempts to purify the active substance against *Xoc* RS105 indicated that it could be a strong polar compound; however, the Tn5-based mutagenesis indicated that a non-ribosomal peptide catalyzed by LgrD may be a major active compound of *P. oryziphila* 1257 because the complementary strain of *lgrD* insertion mutant nearly restored the antibacterial activity to the wild-type levels. In our antiSMASH analysis, the *gntR* and *lgrD* genes that located in the NRPS 4 cluster exhibiting 40% similarity with the AMB biosynthetic gene cluster *ambABCDE* of *P. aeruginosa* PAO1. However, the BLAST analysis in NCBI database showed that the *lgrD* gene encodes a non-ribosomal peptide synthethase of linear gramicidin synthase subunit D. Sequence comparison showed that the gramicidin biosynthetic gene cluster of *lgrABCDT* in *Brevibacillus brevis* displayed 55% similarity with the *lgrD*-related cluster in *P. oryziphila* 1257, and LgrD of *P. oryziphila* 1257 exhibited 43% similarity with the AmbB of *P. aeruginosa* PAO1 and 50% similarity with LgrD proteins of *B. brevis*. These results provide some useful clues for further purification of the target active compound; however, the structure of which needs more verifications from biochemical methods such as high-pressure liquid chromatography and nuclear magnetic resonance. From our Tn5 transposon mutant library, we found that one insertion in the purF, sdhA, dsbB1, serC, and gph genes made 1257 lose the antagonistic activity. Two independent insertions in the *purD* and *purK* genes and one insertion in the *carA*, *purM*, *purC*, and *tuf1* genes reduced the antagonistic properties of 1257 against *Xoc* RS105. Based on the KEGG analyses, we found that these genes were mainly involved in the synthesis of CP and the PPP pathway. Therefore, we speculated that CP and CPR may be the important precursors for synthesis of the activate compounds.

Some studies have shown that most of the bacterial strains applicable for the BLS biocontrol were *Bacillus* strains including *Bacillus amyloliquefaciens*, *B*. *velezensis* and *B*. *cereus* (Zhang et al., 2013; Li et al., 2019; Li S. Z. et al., 2020). For instance, compared to the control treatment, *B*. *amyloliquefaciens* LX-11 significantly reduced the severity of BLS in paddy fields with relative control efficiencies of 60.2% (Zhang et al., 2013). Our results showed that the relative control efficiencies by the *P. oryziphila* 1257 treatments in rice fields and in greenhouses were 53.9 and 66.10%, respectively, which was near to the one by *B*. *amyloliquefaciens* LX-11. However, there are still some problems in practical use, such as tolerance to stress (high temperature in rice-growing season) and stability of *P*. *oryziphila* 1257. We are trying to improve the stress tolerance of *P*. *oryziphila* 1257 through fermentation techniques such as microcapsule bacterial agent to improve its biocontrol effect. Some studies showed that phenazine-1-carboxylic acid (PCA) from *Pseudomonas* species is very effective against *Xoo* and *Xoc* (Xu et al., 2015). PCA as the same name of shenqinmycin has received a pesticide registration certification in China (Jin et al., 2015; Xu et al., 2015). Although *P. oryziphila* 1257 does not produce PCA, there is a long way to go, but it is challenging from purification to commercial use of the antagonistic compounds from *P. oryziphila* 1257 such as PCA. Our results showed that *P. oryziphila* 1257 exhibited specific antagonistic activity against *Xoc* and *Xoo*, whereas whether *P. oryziphila* 1257 is a promising biological control agent for BB needs more evidences from rice field trails.

In summary, we have reported the identification of a novel species, *P. oryziphila*, in the *Pseudomonas* genus. Our results indicated that a non-ribosomal peptide may be one of the major active compounds of *P. oryziphila* 1257 against *Xoc*. Meanwhile, we demonstrated that the type strain *P. oryziphila* 1257^(T)^ is an effective biological control agent for BLS, providing a new microbial resource for biological control of bacterial diseases caused by *X. oryzae*.

## Data Availability Statement

The datasets presented in this study can be found in online repositories. The names of the repository/repositories and accession number(s) can be found in the article/Supplementary Material.

## Author Contributions

RY and SL designed the research. GC supervised the study. YL, YY, and YF analyzed the data and performed part of the biocontrol experiments. RY and LZ wrote the manuscript. RY, SL, YL, YY, YF, LZ, and GC critically revised the manuscript and approved the final version. All authors contributed to the article and approved the submitted version.

## Conflict of Interest

The authors declare that the research was conducted in the absence of any commercial or financial relationships that could be construed as a potential conflict of interest.

## Publisher’s Note

All claims expressed in this article are solely those of the authors and do not necessarily represent those of their affiliated organizations, or those of the publisher, the editors and the reviewers. Any product that may be evaluated in this article, or claim that may be made by its manufacturer, is not guaranteed or endorsed by the publisher.

## Footnotes

1
https://antismash.secondarymetabolites.org/
