# Supplementary material for: Bactericidal Effect of Pseudomonas oryziphila sp. nov., a Novel Pseudomonas Species Against Xanthomonas oryzae Reduces Disease Severity of Bacterial Leaf Streak of Rice
Source: Front Microbiol. 2021 Nov 4;12:759536. doi: 10.3389/fmicb.2021.759536 (PMC8600968; doi:10.3389/fmicb.2021.759536)
Supplement: Supplementary file 1 [file Data_Sheet_1.docx]

***Supplementary Material***

**1. Supplementary Tables**

| **Table S1** **\|** Strains and plasmids used in this study. | | | | |  |  |
| --- | --- | --- | --- | --- | --- | --- |
| **Strains and plasmids** | | **Relevant characteristics** | **Source** | |  |  |
| **Strains** |  | | |  | |  |
| 1257 | The wild type strain of *Pseudomonas. oryziphila* | | | This study | |  |
| CFML 90-83 | The wild type strain of *P. mosselii* | | | CCTCC | |  |
| L48 | The wild type strain of *P. entomophila* | | | CCTCC | |  |
| RS105 | The wild type of *Xanthomonas oryzae* pv. *oryzicola* | | | This lab | |  |
| PXO99^A^ | The wild type of *X. oryzae* pv. *oryzae* | | | ([Salzberg et al., 2008](#_ENREF_3)) | |  |
| RS105-Gus | RS105 carrying a reporter vector pHG1-*hrcC* | | | This lab | |  |
| 100-12 | *lgrD* Tn5 insertion mutant of 1257 | | | This study | |  |
| 56-11 | *carA* Tn5 insertion mutant of 1257 | | | This study | |  |
| 62-42 | *carB* Tn5 insertion mutant of 1257 | | | This study | |  |
| 62-27 | *purF* Tn5 insertion mutant of 1257 | | | This study | |  |
| 93-23 | *serC* Tn5 insertion mutant of 1257 | | | This study | |  |
| *X. campestris* pv. *phaseoli* | Causing bacterial blight of bean | | | This lab | |  |
| *X. axonopodis* pv. *glycines* | Causing bacterial pustule on soybean | | | This lab | |  |
| *X. campestris* pv*. vesicatoria* | Causing pepper spot disease | | | This lab | |  |
| *X. campestris* pv*. malvacearum* | Causing cotton bacterial angular leaf spot | | | This lab | |  |
| *X. campestris* pv*. juglandis* | Causing black spot of walnut | | | This lab | |  |
| *X. axonopodis* pv*. vasculorum* | Causing sugarcane gummosis | | | This lab | |  |
| *X. axonopodis* pv*. allii* | Causing bacterial blight of onion | | | This lab | |  |
| *X. campestris* pv*. musacearum* | Causing banana bacterial wilt | | | This lab | |  |
| *P. syringae* pv*. tomato* DC3000 | Causing bacterial speck on tomato | | | ([Roine et al., 1997](#_ENREF_2)) | |  |
| *Ralstonia solanacearum* | Causing bacterial wilt of plants | | | This lab | |  |
| *Burkholderia glumae* | Causing rice panicle blight | | | This lab | |  |
| *Acidovorax citrulli*. | Causing watermelon bacterial fruit blotch | | | This lab | |  |
| *Magnaporthe oryzae* | Causing rice blast | | | This lab | |  |
| *Fusarium graminearum* | Causing fusarium head blight | | | This lab | |  |
| *F. oxysporium* | Causing root rot disease of tomato | | | This lab | |  |
| *Botrytis cinerea* | Causing tomato gray mold disease | | | This lab | |  |
| *Phytophthora capsici* | Causing phytophthora blight of Pepper | | | This lab | |  |
| **Plasmids** | | | | |  |  |
| EZ-Tn5 <R6Kγori/KAN-2> | Containing Tn5 transposon, Km^r^ | | | Epicentre | | |
| pML123 | Broad- host- range vector, Gm^r^ | | | ([Labes et al., 1990](#_ENREF_1)) | | |

**Note**: Km^r^, kanamycin resistance, Gm^r^, gentamycin resistance

**Table S2 |** Primers used in this study.

| **Primers** | **Sequence 5’ to 3’** | **Description** |
| --- | --- | --- |
| 27F | AGAGTTTGATCCTGGCTCAG | Universal primers used for amplifying the bacterial *16SrRNA* gene |
| 1492R | TACGGCTACCTTGTTACGACTT |  |
| pML123-F | AGGTTGGGAAGCCCTGCAAA | Primers for confirmation of the pML123-based construct |
| pML123-R | TAGCAGCCAGTCCCTTCCCGCTTCA |  |
| *carA*-F | CGCGGATCCTTGACAAAGVVAGCCATACT | Primers for functional complementation of the *carA* insertion mutant |
| *carA*-R | TGCTCTAGATCAGCGGCGCTTGGCCATGG |  |
| *carB*-F | CGCGGATCCATGCCAAAACGTACAGACATCAAAA | Primers for functional complementation of the *carB* insertion mutant |
| *carB*-R | TGCTCTAGATCATGCTTTAAGTCCTGCATG |  |
| *serC*-F | CGCGGATCCGCAGCAAGAGTAAGCAAGTG | Primers for functional complementation of the *serC* insertion mutant |
| *serC*-R | CGAGCTCTCAGCCGTGCTCCTTCTCGA |  |
| *purF*-F | CCCAAGCTTGGGGGCAATGCTCAACTTCC | Primers for functional complementation of the *purF* insertion mutant |
| *purF*-R | CGAGCTCTCAGTTGTTGTACAGGTCGA |  |
| *lgrD*-F | GCTCTAGAACTGGCAGGCCGCGCCAG | Primers for functional complementation of the *lgrD* insertion mutant |
| *lgrD*-R | CCCAAGCTTTCAGATGAAGCCGATGTCGGC |  |
| Tn5-F | GACAATCTATCGATTGTATGGGAA | Specific primers used for Tn5 transposon sequencing |
| Tn5-R | GTCAGCGTAATGCTCTGCCAGTGT |  |

**Note**: Underlined bases indicate restriction enzyme sites

**Table S3 |** Physiological and biochemical characteristics of *P. oryziphila* 1257 (*Po*), *P. entomophila* (*Pe*) L 48 and *P. mosselii* (*Pm*) CFMT90-83.

| **Characteristics** | ***Po***  **1257** | ***Pe***  **L48** | ***Pm***  **CFML 90-83** |
| --- | --- | --- | --- |
| **Assimilation of (API 20NE):** |  |  |  |
| Reduction of nitrate | - | - | **-** |
| Indole | - | - | **-** |
| Acidize of glucose | - | - | **-** |
| Arginine dihydrolase | + | + | **+** |
| Urease | **-** | **-** | **+** |
| Esculin hydrolysis(β-D-Glucosidase) | - | - | **-** |
| Hydrolysis of gelatin | + | + | **+** |
| β-galactosidase | - | - | **-** |
| Glucose | + | + | **+** |
| Arabinose | - | - | **-** |
| Mannose | + | + | **+** |
| Mannitol | + | + | **+** |
| N-acetyl-d-glucosamine | - | + | + |
| Maltose | - | - | **-** |
| Gluconate | + | + | **+** |
| Decanoic acid | + | + | **+** |
| Adipic acid | - | - | **-** |
| Malic acid | + | + | **+** |
| Citric acid | + | + | **+** |
| Phenylacetic acid | + | + | **+** |
| **Acid from (API 20E):** |  |  |  |
| α-D- Glucose | + | + | + |
| D-Melibiose | - | - | - |
| L-Arabinose | + | + | + |
| **Assimilation of (50 CH):** |  |  |  |
| Glycogen | - | W | **-** |
| Tween 80 | W | + | **+** |
| D-Arabitol | - | + | **+** |
| L- Fucose | W | W | - |
| Psicose | - | - | + |
| Dimethyl succinate | + | + | W |
| Acetic acid | W | + | **+** |
| D- (+)-Glucuronic acid | + | - | **-** |
| α-Ketobutyric acid | + | + | W |
| Bromosuccinic acid | W | + | **+** |
| Thymidine | W | - | **-** |
| 2,3-Butanediol | - | + | **+** |
| Glucose-1-phosphate | W | - | **-** |

Note: + indicates positive reaction, - indicates negative reaction, W indicates weakly positive reaction

**Table S4 |** The type IV pilus (T4P) system associated genes in *P. oryziphila* 1257

| **Gene**  **ID** | **Protein name** | **Function** | ***Pe***  **L48^a^** | ***Pm***  **CFML90-83^b^** |
| --- | --- | --- | --- | --- |
| *chr_orf 01104* | FimT | Putative minor pilin protein | 74.20% | 72.50% |
| *chr_orf 01108* | PilE | Putative minor pilin protein | Non | 81.80% |
| *chr_orf 01126* | PilD | Pre-pilin peptidase | Non | Non |
| *chr_orf 01127* | PilC | Putative T4P assembly motor protein | Non | Non |
| *chr_orf 01128* | PilA | Major pilin protein | Non | Non |
| *chr_orf 02434* | PilZ | Putative T4P assembly protein | 91% | 90% |
| *chr_orf 05629* | PilL | Putative T4P associated protein | Non | 89% |
| *chr_orf 05630* | PilN2 | The Type IVB alignment subcomplex protein | Non | 96% |
| *chr_orf 05631* | PilO | The Type IVB alignment subcomplex protein | Non | 89% |
| *chr_orf 05632* | PilQ2 | The outer membranes pore complex protein | Non | 94% |
| *chr_orf 05633* | TcpE | Toxin coregulated pilus biosynthesis protein | Non | 96% |
| *chr_orf 05634* | PilS | Sensor protein of two-component system | Non | 99% |
| *chr_orf 05635* | PilT2 | Putative T4P assembly motor protein | Non | 97% |
| *chr_orf 05636* | PilV | Putative minor pilin protein | Non | 83% |
| *chr_orf 06919* | PilJ | Putative T4P methyl-accepting chemotaxis transducer | Non | Non |
| *chr_orf 06130* | PilF | Putative T4P associated protein | 93% | 90% |
| *chr_orf 07091* | PilQ1 | The outer membranes pore complex protein | 95% | 90% |
| *chr_orf 07092* | PilP | The alignment subcomplex protein | 70% | 64% |
| *chr_orf 07093* | PilN1 | The alignment subcomplex protein | 68% | 66% |
| *chr_orf 07094* | PilM | The alignment subcomplex protein | 68% | 66% |
| *chr_orf 07110* | PilT1 | Putative T4P assembly motor protein | 89% | 88% |

Non indicated that the gene product was not identified.

^a^ indicates sequence similarity of gene products between *P. oryziphila* 1257 and *P. entomophila* (*Pe*) L48, and ^b^ indicates sequence similarity of gene products between *P. oryziphila* 1257 and *P. mosselii* (*Pm*) CFMT90-83.

**TABLE S5|** Genes and gene products potentially involved in secondary metabolites in *P. oryziphila* 1257

| **Category** | **Gene name** | **Gene function** | ***Pe***  **L48^a^** | ***Pm***  **CFML90-83^b^** |
| --- | --- | --- | --- | --- |
| **Toxins** | *aprA* | alkaline metalloprotease AprA | 95.00% | 94.00% |
|  | *rtx* | RTX toxin | 76.00% | 74.00% |
| **Antibiotics** | *hcnA* | hydrogene cyanide synthase HcnC | 97.00% | 97.00% |
|  | *hcnB* | hydrogene cyanide synthase HcnB | 96.00% | 95.00% |
|  | *hcnC* | hydrogene cyanide synthase HcnA | 93.00% | 95.00% |
| **Cyclic lipopeptides** | *lgrD* | linear gramicidin synthase subunit D | 87.00% | 86.00% |
|  | *iacR* | putative IAA degradation enzymes, an ABC transporter | 89.54% | 89.37% |
|  | *paa* | phenylacetic acid degradation protein | 92.00% | 91.00% |
| **Plant–bacterial interactions** | *ydjL* | 2,3-butanediol dehydrogenase | Non | Non |
|  | *acoB* | acetoin dehydrogenase subunit beta | 86.5% | 84.60% |
|  | *pqqE* | pyrroloquinoline quinone biosynthesis protein PqqE | 97.00% | 97.00% |
|  | *pqqD* | pyrroloquinoline quinone biosynthesis protein PqqD | 98.00% | 98.00% |
|  | *pqqB* | pyrroloquinoline quinone biosynthesis protein PqqB | 99.00% | 98.00% |

Non indicated that the gene product was not identified.

^a^ indicates sequence similarity of gene products between *P. oryziphila* 1257 and *P. entomophila* (*Pe*) L48, and ^b^ indicates sequence similarity of gene products between *P. oryziphila* 1257 and *P. mosselii* (*Pm*) CFMT90-83.

**Table S6 |.** Secondary metabolite biosynthesis gene clusters of *P. oryziphila* 1257 predicted by antiSMASH.

| **Cluster** | **Type** | **Location** | **Most similar known cluster (%)**^a^ |
| --- | --- | --- | --- |
| NAGGN | dipeptide | 1,551,112-1,565,974 | ND |
| Bac 1 | Bacteriocin | 2,017,823-2,027,234 | ND |
| NRPS 1 | siderophores | 2,371,708-2,445,814 | Pyoverdin biosynthetic gene cluster (13%) |
| Bac 2 | Bacteriocin | 3,587,222-3,598,055 | ND |
| NRPS 2 | siderophores | 3,728,418-3,799,305 | Cupriachelin biosynthetic gene cluster (35%) |
| NRPS 3 | siderophores | 4,150,636-4,203,580 | Pyoverdin biosynthetic gene cluster (11%) |
| APE | Aryl polyene | 5,672,348-5,715,955 | Arylpolyene (APE) biosynthetic gene cluster (35%) cluster |
| NRPS 4 | NRPS-like | 5,865,882-5,909,313 | L-2-amino-4-methoxy-trans-3-butenoic acid (AMB) biosynthetic gene cluster (40%) |

^a^ indicates sequence similarity of gene cluster between *P. oryziphila* 1257 and the best BLAST hit from the antiSMASH database.

ND indicates that similar gene cluster was not detected in the antiSMASH database.

**TABLE S7** **|** Genes involved in *P. oryziphila* 1257 antibacterial activity against *Xoc* RS105

| **Gene ID** | **Gene name** | **Gene function** | ***Pe***  **L48^d^** | ***Pm***  **CFML90-83^e^** |
| --- | --- | --- | --- | --- |
| *chr_orf 01014* | *carA*^b^ | Carbamoyl-phosphate synthase small chain | 99.00% | 99.00% |
| *chr_orf 01015* | *carB*^a^ | Carbamoyl-phosphate synthase large chain | 99.00% | 99.00% |
| *chr_orf 01784* | *purM*^b^ | Phosphoribosylformylglycinamidine cyclo-ligase | 90.00% | 89.00% |
| *chr_orf 02193* | *purF*^a^ | Amidophosphoribosyltransferase | 99.00% | 99.00% |
| *chr_orf 05580* | *purC*^b^ | Phosphoribosylaminoimidazole-succinocarboxamide synthase | 95.22% | 95.64% |
| *chr_orf 05904* | *purL*^a^ | Phosphoribosylformylglycinamidine synthase | 88.00% | 87.00% |
| *chr_orf 06665* | *purD*^b^ | Phosphoribosylamine glycine ligase | 98.00% | 99.00% |
| *chr_orf 07447* | *purK^b^* | N5-carboxyaminoimidazole ribonucleotide synthase | 87.00% | 86.00% |
| *chr_orf 07326* | *gntR*^b^ | GntR family transcriptional regulator | 96.00% | 95.00% |
| *chr_orf 07332* | *lgrD*^b^ | Non ribosomal peptide synthethase, loading module; Linear gramicidin synthase subunit D | 87.00% | 86.00% |
| *chr_orf 05131* | *sdhA*^a^ | Succinate dehydrogenase flavoprotein subunit | 91.00% | 91.00% |
| *chr_orf 06235* | *dsbB1*^a^ | Disulfide bond formation protein B1 | 80.00% | 78.00% |
| *chr_orf 00661* | *tuf1*^b^ | GTPases - translation elongation factor Tu | 99.00% | 99.00% |
| *chr_orf 06227* | *gph*^a^ | Phosphoglycolate phosphatase | 77.00% | 40.30% |
| *chr_orf 01975* | *serC*^a^ | Phosphoserine aminotransferase | 99.00% | 98.00% |
| *chr_orf 01009* | *dnaK*^c^ | Chaperone protein DnaK | 98.00% | 98.00% |
| *chr_orf 01562* | *argG*^c^ | Argininosuccinate synthase | 99.00% | 99.00% |
| *chr_orf 04535* | *sohB*^c^ | Probable protease SohB; Periplasmic serine proteases (ClpP class) | 79.00% | 81.00% |
| *chr_rRNA2* | *rRNA*2^c^ | 23s_rRNA | 99.00% | 99.00% |

^a, b, c^ genes associated with absolutely losing, apparently and partially attenuated antagonistic activity against *Xoc* RS105, respectively.

^d^ indicates sequence similarity of gene products between *P. oryziphila* 1257 and *P. entomophila* (*Pe*) L48, and ^e^ indicates sequence similarity of gene products between *P. oryziphila* 1257 and *P. mosselii* (*Pm*) CFMT 90-83.

**2. Supplementary Figures**

**
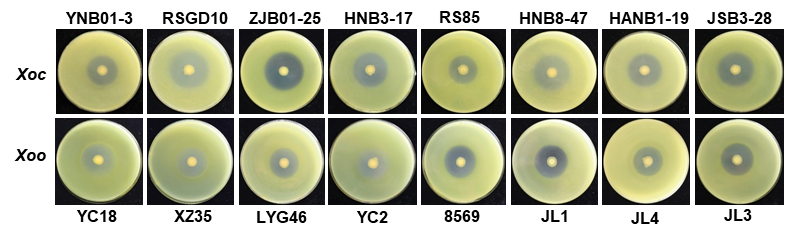
**

**Figure S1.** **Antibacterial activity assays of *P.* *oryziphila* 1257 against *Xoc* and *Xoo* strains.** *Xoc* strains including YNB01-3, RSGD10, ZJB01-25, HNB3-17, RS85, HNB8-47, HANB1-19, JSB3-28. *Xoo* strains including YC18, XZ35, LYG46, YC2, 8569, JL1, JL4, JL3.


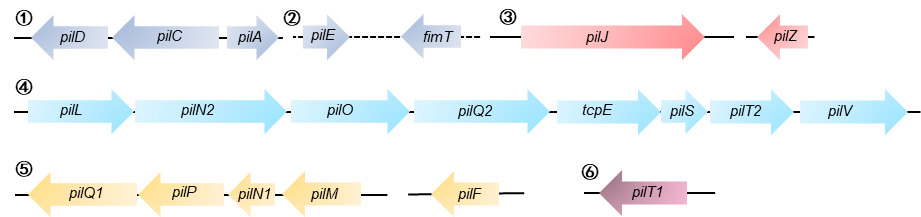


**Figure S2.** **Map of the type IV pilus system (T4P) related genes or gene clusters in *P.* *oryziphila* 1257 genome.** The dashed lines indicate that genes are not adjacent in the cluster.


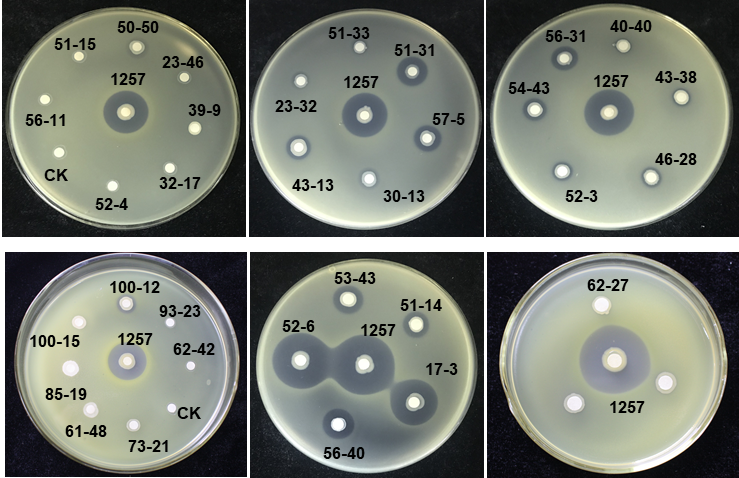


**Figure S3.** **Antagonistic activity of 32 mutants against *Xoc* RS105.** *P.* *oryziphila* 1257 as the wild type strain and CK as the negative control.


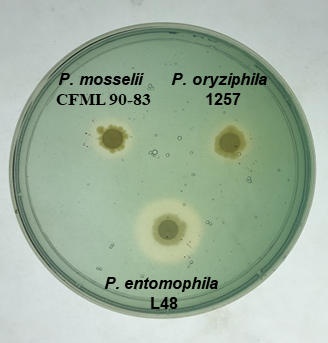


**Figure S4. The chrome azurol S (CAS) plates assay** of *P.* *oryziphila* 1257, *P. entomophila* L48 and *P. mosselii* CFML 90-83.
